# Low dose photodynamic therapy harmonizes with radiation therapy to induce beneficial effects on pancreatic heterocellular spheroids

**DOI:** 10.18632/oncotarget.26780

**Published:** 2019-04-05

**Authors:** Anne-Laure Bulin, Mans Broekgaarden, Diane Simeone, Tayyaba Hasan

**Affiliations:** ^1^ Wellman Center for Photomedicine, Department of Dermatology, Harvard Medical School and Massachusetts General Hospital, Boston, MA, USA; ^2^ Department of Surgery, NYU Langone Health, New York, NY, USA; ^3^ Perlmutter Cancer Center, NYU Langone Health, New York, NY, USA; ^4^ Department of Pathology, NYU Langone Health, New York, NY, USA

**Keywords:** low dose photodynamic therapy, radiation therapy, spheroids, pancreatic cancer

## Abstract

Photodynamic therapy (PDT) has seen long standing interest as a therapy for resistant cancers, but the main Achilles’ heel for its successful clinical exploitation is the use of poorly penetrating visible light. This limitation could be overcome by using radioluminescent nanoparticles, which can be excited during radiation therapy (RT) with penetrating X-rays. When infused in tumors, X-ray activated-nanoscintillators act as internal light sources and excite nearby photosensitizers. Recent studies demonstrated that it is realistic to achieve low dose PDT with current nanoscintillators. However, as the origin of enhanced RT efficacy with nanoscintillators may have varying origins, we aimed to answer the basic question: Is a combination of low-dose PDT beneficial to the RT efficacy in clinically relevant models of cancer?

Pancreatic cancer (PanCa) remains a lethal disease for which RT is part of the palliative care and for which PDT demonstrated promising results in clinical trial. We thus evaluated the combination of low-dose PDT and RT delivered in absence of nanoscintillators on various heterocellular spheroid models that recapitulate the clinical heterogeneity of PanCa. Although therapeutic effects emerged at different timepoints in each model, the RT/PDT combination uniformly achieved favorable outcomes. With RT providing stunted tumor growth while PDT drove adjuvant apoptotic and necrotic cell death, the combination produced significantly smaller and less viable PanCa spheroids.

In conclusion, the beneficial RT/PDT treatment outcomes encourage the further development of nanoscinitillators for X-ray-activated PDT. Assessment of such combination treatments should encompass multiparametric and temporally-spaced assessment of treatment effects in preclinical cancer models.

## INTRODUCTION

Pancreatic cancer (**PanCa**) remains one of the most lethal types of cancer for which 5-year survival rates do not exceed 6%. Currently, the only curative option encompasses a complete surgical resection for which only 15 to 20% of the patients are eligible [1]. However, the 5-year survival rate for patients undergoing surgery with curative intent remains limited to 20% due to disease recurrence [2]. Although adjuvant chemotherapy or radiation therapy (**RT**) are usually involved in the standard-of-care, they do not overcome the poor prognosis [1, 3]. Chemotherapy [4] or RT [5, 6] are also employed as palliative care for inoperable patients, yet no beneficial effects on long term survival rates have been reported, underscoring a clear need for new therapies.

Recently, photodynamic therapy (**PDT**), a light-activated cancer therapy has demonstrated promise for the treatment of PanCa in both preclinical and clinical trials [7, 8]. PDT relies on the light activation of non-toxic photosensitizers (**PS**) that induces photochemical reactions culminating in the generation of reactive molecular species (**RMS**). These RMS trigger highly localized oxidation reactions in the tumor tissue, resulting in massive cell death, mesoscopic effects such as tumor hypoxia and nutrient starvation, as well as abscopal effects by initiating an anti-tumor immune response [9–15]. However, PDT is intrinsically limited by the shallow penetration of light in tissues [12, 16]. Although percutaneous placement of optical fibers is possible, the irradiated volume remains restricted and makes the procedure highly invasive and prone to complications [16].

Theoretically, this major limitation of PDT can be overcome with the use of scintillating nanoparticles, which potentially allow the activation of PDT within deep seeded or large tumor volumes [17, 18]. Scintillating nanoparticles down-convert ionizing radiations, including X-rays, into many lower energy photons with energy in the visible range [19]. If conjugated to PS and accumulated in a tumor before RT, nanoscintillators may be used as local light sources to subsequently excite proximal PS, thus overcoming the light penetration issues commonly associated with conventional PDT in a non-invasive way. Encouraging results have been obtained by us and others during proof-of-concept studies performed *in silico*, *in vitro* and *in vivo*, emphasizing the promise of this approach [17, 20–32].

However, these encouraging studies did not account for the plethora of potential mechanisms to explain the observed *in vitro* and *in vivo* findings of the nanoscintillator-mediated X-ray PDT. The use of heavy-metal nanoparticles complicates the interpretation of such results as the auxiliary therapeutic benefits may stem from radiation dose-enhancement by the heavy metal nanoparticles [33], phototoxicity or photobiomodulation of the nanoscintillator-emitted light [34]. A second shortcoming relates to the use of biological models that do not recapitulate the heterogeneity that typifies cancer tissues in clinical settings. To fully understand the promise of X-ray activated PDT during RT using nanoscintillators, it is therefore imperative to first evaluate the RT/PDT combination in absence of heavy-metal nanoparticles on models with diverse genotypes and phenotypes.

In this study, we therefore investigated whether PDT synergizes with RT when combined in the absence of nanoparticles. Studies published by us and others demonstrated that the energy transfer between nanoscintillators and conjugated PS will likely achieve moderate-to low-dose PDT [35–37]. Thus, in the current study, we explored a combination of RT with low dose PDT to evaluate whether this achieved beneficial effects in the treatment of PanCa, which represents a clinically relevant disease for which RT is involved in the standard of palliative care. We established various phenotypically distinct three-dimensional (**3D**) cancer cultures that additionally comprised stromal partner cells, to be used as a platform for the evaluation of RT/PDT treatment responses in a translationally relevant manner.

## RESULTS

### Characterization of the three pancreatic tumor models comprising genotypically and phenotypically distinct cancer cell types

In order to incorporate some of the *in vivo* features into an *in vitro* model, we grew spheroids as 3D models of tumor. These models are gaining increasing interest as they bridge a gap between monolayer-grown cellular cultures and *in vivo* models: they recapitulate more faithfully the tumor microenvironment than 2D cultures without trading of the high throughput aspect [38–44]. In order to represent the heterogeneity of PanCa that is observed in clinic, we established three distinct models consisting of either MIA PaCa-2, AsPC-1 or Capan2 cells. As a dense fibrotic stroma has been identified as a clinical hallmark of PanCa [45–49], that associates with treatment resistance [50–54], pancreatic cancer associated fibroblasts (pCAF) were incorporated into the spheroids [55, 56].

With respect to the chosen cancer cell lines, both MIA PaCa-2 and Capan2 cells were originally derived from a primary tumor whereas AsPC-1 cells were derived from PanCa ascites, thus both primary and metastatic tumor models have been investigated [57]. Together, these cell lines additionally represent genetically distinct cell types that are frequently observed in the clinic: while all three cell lines express the KRAS mutation, which is found in almost 100% of PanCa patients [1], only MIA PaCa-2 and AsPC-1 cells present a TP53 mutation, a mutation reported in about 50% of the patients, usually when they present a late stage disease. Although p53 mutations are often associated to a higher resistance to treatment including radiation therapy, the correlation between p53 mutation and radio-resistance is not that simple and in some cases an inhibition of p53 even showed some beneficial effect on cell survival post-treatment, emphasizing the importance and complexity of this pathway in driving treatment sensitivity [58]. Thus, although there is no direct correlation between the p53 status of a cell line and its treatment susceptibility, it is a determinant parameter to consider.

**Figure 1:**
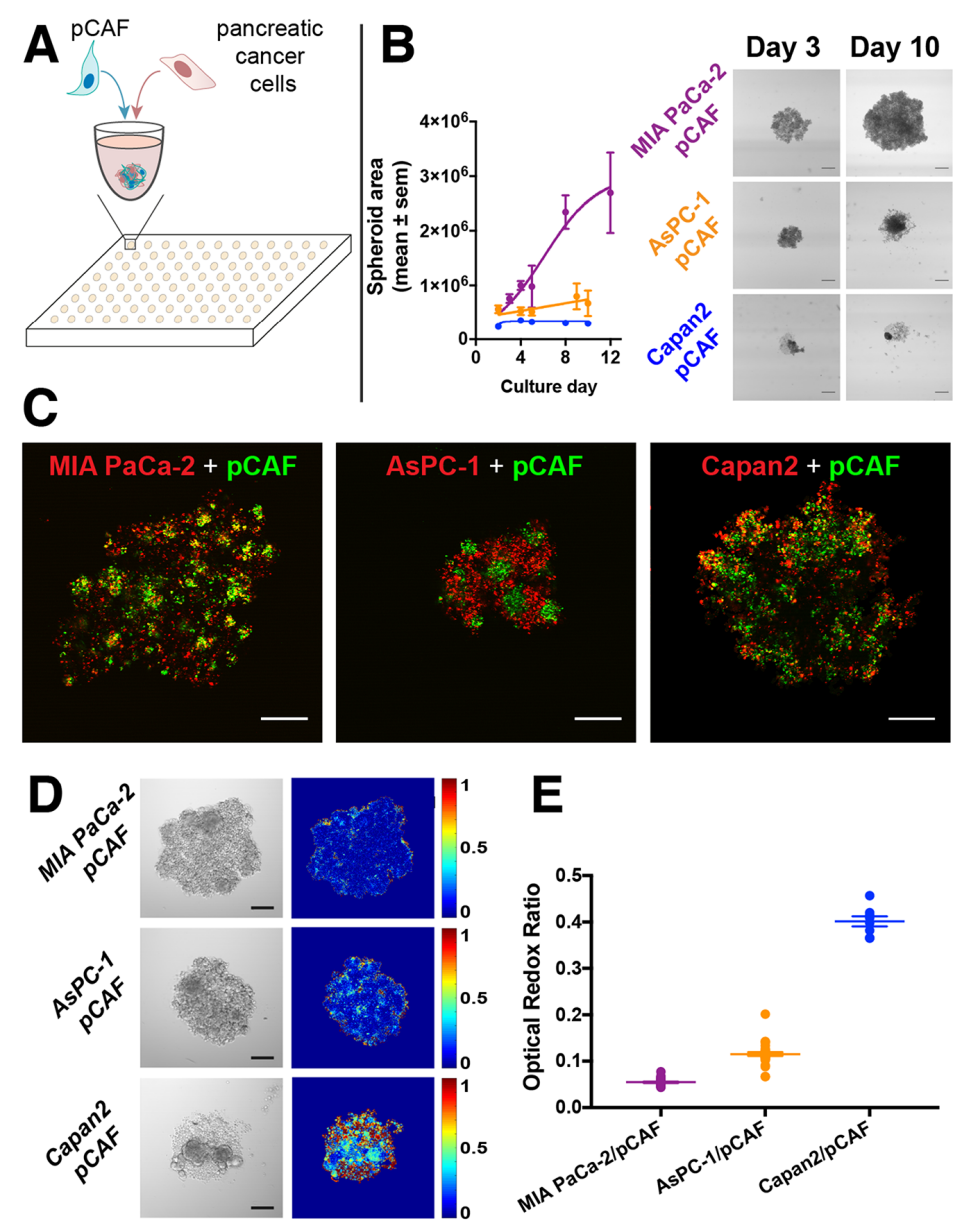
**(A)** Pancreatic cancer cell lines (MIA PaCa-2, Capan2 and AsPC-1) were co-cultured with patient derived pancreatic cancer associated fibroblasts (pCAF) in U bottom, ultra-low adhesion 96-well plates. **(B)** After cell seeding, the spheroids were regularly imaged (scale bar: 400μm) and their surface was plotted (in μm^2^) as a function of the time. MIA PaCa-2/pCAF culture is displayed in purple, the AsPC-1/pCAF in orange and the Capan2/pCAF in blue. **(C)** A staining assay leveraging the exceptionally high 2-photon absorption cross-section of quantum dots was developed to identify, here on day 2, the internal architecture of the spheroids and investigate the relative disposition of each cell type: PanCa cell line being depicted in red while pCAF are represented in green (Scale bar: 200μm). In both MIA PaCa-2/pCAF and AsPC-1/pCAF models, the pCAF form dense pockets surrounded by PanCa cell lines. However, in the case of Capan2/pCAF cultures, the two cell lines are more homogeneously distributed within the culture. **(D)** Representative bright-field images (scale bar = 200μm) and Optical Redox Ratio (ORR) heatmaps of untreated spheroids, measured on day 3. **(E)** Quantification of the spheroids ORR (mean ± sem) assessed on day 3 (no treatment applied).

Characterization of tumor spheroid growth revealed that the MIA PaCa-2/pCAF spheroids display logistic growth kinetics. In contrast, the AsPC-1/pCAF and the Capan2/pCAF remain significantly smaller and rapidly reach a plateau in size. However, despite that the spheroid core remains unchanged, it can be observed that cells are escaping the dense core to form a loose spheroid periphery (Figure 1B, day 10). Fluorescent labeling of the individual cell populations enabled the investigation of the intranodular distribution of the PanCa cells and pCAF. In both MIA PaCa-2/pCAF and AsPC-1/pCAF cultures, the pCAF form dense pockets surrounded by the PanCa cell lines. In the case of the Capan2/pCAF culture, the two cell lines seem more homogeneously distributed within the spheroid.

### Selection of PDT and RT treatment parameters for establishing the RT/PDT combination

We investigated the susceptibility of the different PanCa models to BPD-PDT, focusing on the effects of treatment on (1) normalized spheroid size, and (2) intensity of the necrotic staining (Supplementary Figure 1A, 1C and 1E). Regarding the normalized spheroid area, a negative correlation was observed between the MIA PaCa-2/pCAF spheroids area and the PDT dose. However, the Capan2/pCAF and AsPC-1/pCAF cultures did not exhibit the same trend: while the overall area stayed stable for the Capan2/pCAF culture, it slightly increased for the AsPC-1/pCAF spheroids. In addition, it should be noted that for the AsPC-1/pCAF spheroid, it is not the size of the spheroids core that is increasing but the spread of the cells escaping the core, as it can be seen on the bright-field images. All three culture-types showed a positive correlation between the PI fluorescence intensity and the PDT light dose (Supplementary Figure 1A, 1C and 1E). Using the dose response regression curves, we extracted a low-killing light dose of PDT that was further used for the combination treatment: 2.5J/cm^2^.

In parallel, we investigated RT dose-response effects on the various spheroid models using doses ranging from 1Gy to 20Gy (Supplementary Figure 1B, 1D and 1F). The radiation dose range was chosen based on previous studies performed either on monolayer cultures or on spheroids [50, 59–62]. When measured on day 5, neither the spheroid area nor the PI intensity changed significantly with increasing doses. However, when investigated at later time points (day 8 or day 12), the MIA PaCa-2/pCAF spheroids area strongly decreased with time for increasing RT doses (Supplementary Figure 2A). For further studies, three doses of RT (2Gy, 10Gy and 20Gy) were systematically investigated.

### RT as a spheroid growth inhibitor

**Figure 2:**
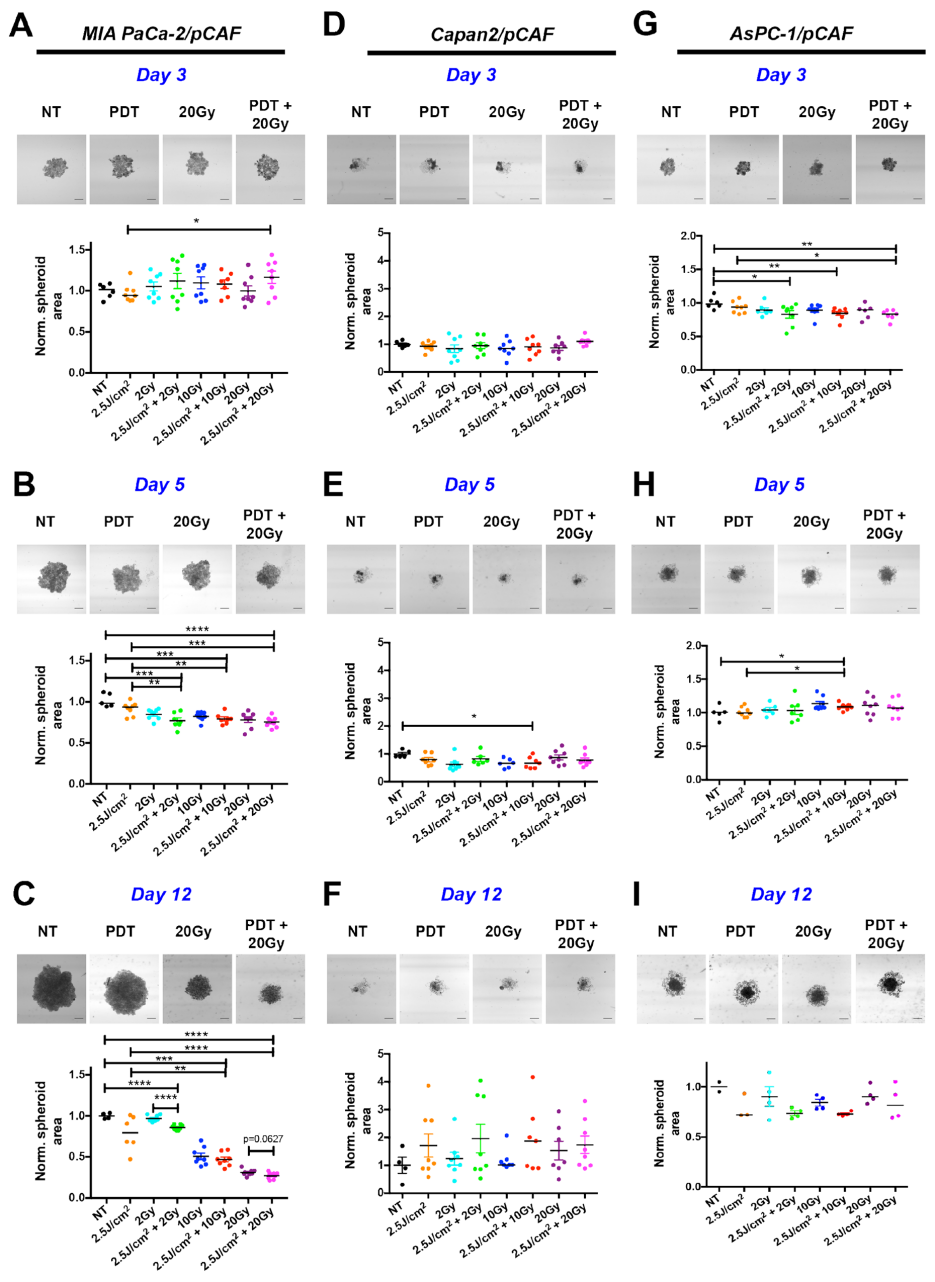
Longitudinal monitoring of the spheroids area as a function of the treatment condition For each time point and culture type, four representative images are presented corresponding to a control (*NT*), 2.5J/cm^2^ PDT (*PDT*), 20 Gy RT and a combination of 2.5J/cm^2^ PDT + 20Gy RT (PDT+20Gy) (Scale bar: 400μm). Results are presented for MIA PaCa-2/pCAF (**A**, **B** and **C**), Capan2/pCAF (**D**, **E** and **F**) and for AsPC-1/pCAF (**G**, **H** and **I**) and were assessed on day 3 (**A**, **D**, **G**), day 5 (**B**, **E**, **H**) and day 12 (**C**, **F**, **I**). For each condition, a scatter plot represents the area of each individual spheroids normalized on the average area of the untreated spheroids together with the median value of each treatment group. A strong decrease in the spheroid area is reported for the MIA PaCa-2/pCAF cultures for each treatment. The effect gets stronger at day 12, i.e. 9 days post treatment. For this time point, a mild beneficial effect is reported when combining 20Gy RT with PDT. For both Capan2/pCAF and AsPC-1/pCAF cultures the size variations are slighter regardless of the treatment and the day of measurement.

Based on the aforementioned dose-escalation experiments, a combination therapy of PDT (2.5J/cm^2^) and RT (2Gy, 10Gy, and 20Gy) was designed. We first investigated the effect of this combination treatment on the growth of the spheroids. For the MIA PaCa-2/pCAF co-cultures, while we observe divergent sizes on day 3 (Figure 2A), spheroid sizes gradually decrease on day 5 (Figure 2B) and day 12 (Figure 2C) in a RT dose-dependent manner. On day 12 the spheroids that received RT continued to diminish, reaching an area that was ∼25% of the untreated controls for the 20Gy RT dose. When spheroids received both PDT and RT compared to RT alone, there was a slight additional reduction in spheroid size, regardless of the RT dose.

As for the Capan2/pCAF cultures, spheroid sizes (including spheroid cores and the cell halo) are notably more heterogeneous. Regardless of the given treatment, the spheroid size does not significantly vary (Figure 2D, 2E and 2F) and the combination RT/PDT does not demonstrate any noticeable benefit on PDT or RT based on size alone.

An intermediate situation is observed for the AsPC-1/pCAF cultures: on day 3 the spheroids form a homogeneous mass (Figure 2G), whereas a halo of cells starts appearing around the cores on day 5 regardless of the treatment group (Figure 2H), which is still present on day 12 (Figure 2I). As for the normalized area, no noticeable changes are reported on day 3 and 5. However, some differences appear on day 12 where the spheroids that were treated with a RT/PDT combination have smaller cores than the spheroids that were exposed to RT (Figure 2I). However, when reporting the total area (core+halo), we observe a size increase for the spheroids that received the RT/PDT combination (Supplementary Figure 3), which was caused by an increase in the loose cellular halo, potentially caused by degradation of the spheroid core integrity or increased cellular migration. Similar phenomenon of spheroid disintegration was reported for ovarian cancer organoids following PDT treatment [63].

### PDT uniformly drives the necrotic response during RT/PDT treatment

Whereas the spheroid area gives an indication about the ability of the treatment to control the tumor size, it does not convey any information about the health of the remaining cell population. We therefore performed a live/dead fluorescence staining to identify both necrotic and viable cells within the spheroids [4, 64].

**Figure 3:**
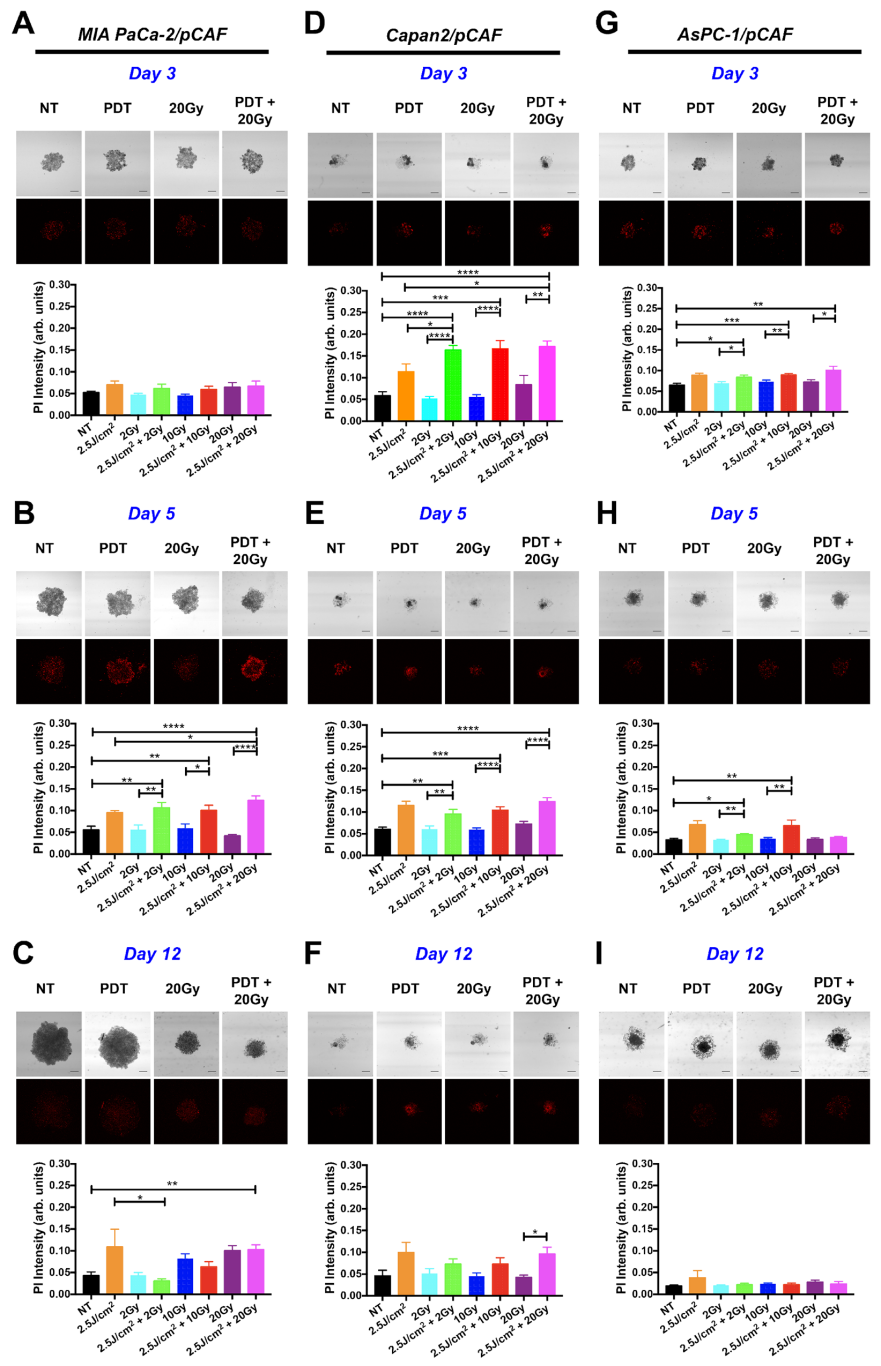
Necrotic population generated in the spheroids by individual or combination treatments For each type of culture: MIA PaCa-2/pCAF **(A**, **B**, and **C)**, Capan2/pCAF **(D**, **E**, and **F)** or AsPC-1/pCAF **(G**, **H**, and **I)**, a PI-staining protocol was performed in order to assess the necrotic population using subsequent confocal microscopy; those assays were performed on day 3 (4 hours post treatment; **A**, **D** and **G**), on day 5 (2 days post-treatment; **B**, **E** and **H**) or on day 12 (9 days post treatment; **C**, **F** and **I**). For each condition, a representative PI fluorescence image is presented side-by-side with the associated bright-field image (Scale bar= 400μm) for a few selected conditions: the untreated control (NT), the PDT alone (2.5J/cm^2^), a 20 Gy RT alone, and a 20Gy RT combined with a 2.5J/cm^2^ PDT (PDT+20Gy). For each condition, quantitative data is represented on bar graphs where the PI fluorescence intensity averaged over the entire spheroid area is plotted for each treatment condition. For each group, N>6 spheroids coming from at least 2 biological repeats; the data represents the mean +/- standard error.

For the MIA PaCa-2/pCAF spheroids, no treatment effects were detected within 4h post-treatment (day 3, Figure 3A), but prominent effects emerged after 48h (day 5, Figure 3B). At this time point, there was a significant increase in the extent of necrosis in all groups receiving PDT. The extent of necrosis was highest in the 2.5J/cm^2^ PDT+20Gy RT combination compared to the group that received only the identical dose of PDT. In contrast, no increase in necrosis was observed in spheroids receiving RT alone. On day 12 (Figure 3C), the extent of necrosis remained high in the group receiving PDT alone, whereas a RT-dose-dependent increase in necrosis was detected (although not indicated on the figure, the difference measured between the PI intensity for the 2Gy group and 10Gy or 20Gy is statistically significant: p=0.0082 and p=0.0004 respectively). When comparing these findings with the effects of treatment on spheroid size (Figure 2), it can be observed that while PDT induces necrosis, it is ineffective in reducing the overall size of the spheroids. In contrast, RT alone effectively hampers spheroid growth in absence of necrosis. The major beneficial effect of the RT-PDT combination treatment therefore stems from the impaired growth by RT and increased necrosis by PDT, together resulting in the most effective reduction in size on day 12 for the 2.5J/cm^2^+20Gy regimen (Figure 2C).

As for the Capan2/pCAF cultures, the treatment effects could be effectively detected within the first 4h post-treatment (day 3, Figure 3D). Similar to the MIA PaCa-2/pCAF spheroids, albeit at a different time point, PDT facilitates a significant increase in the extent of necrosis in the spheroids receiving either PDT alone or the RT/PDT combinations. Again, there was no increase in necrosis in spheroids receiving RT alone. The PDT-induced necrotic response remains significantly elevated on day 5 (Figure 3E), but gradually diminishes over time until day 12 (Figure 3F). A close inspection of the necrosis images indicates that the dense spheroid cores display the highest degrees of necrosis. Therefore, although the spheroid areas were increased by the PDT-RT combination, the majority of the spheroids are necrotic. The increased halo may stem from reduced cell-cell adhesion and increased escape from the necrotic spheroid cores. Taken together, as similar beneficial effects of PDT on the RT efficacy as observed in the MIA PaCa-2/pCAF spheroids are reported in the Capan2/pCAF cultures, we can conclude that PDT promotes the induction of necrosis that is otherwise not induced by RT alone (Figure 3D).

With respect to the AsPC-1/pCAF spheroids, similar effects as described for the Capan-2/pCAF spheroids are observed, albeit to a lesser extent. A significant increase in the extent of necrosis can be detected shortly after treatment on day 3 (Figure 3G), whereas these effects are diminished at later time points (Figure 3H and 3I). When taking into account the total spheroid size at later time points, we can discern a comparable increase in the spheroid halos in cultures exposed to the PDT-RT combinations. A similar response resulting from the induction of early necrosis and a prolonged reduction in cell-cell adhesion as observed in the Capan-2/pCAF cultures can thus be observed in the AsPC-1/pCAF spheroids. The reduced efficacy of the treatments may be attributed to the highly resistant nature of this cell line to cancer therapies [65].

### RT/PDT combination reduces spheroid size more than each individual treatment and increase necrosis in a more than additive manner

**Figure 4:**
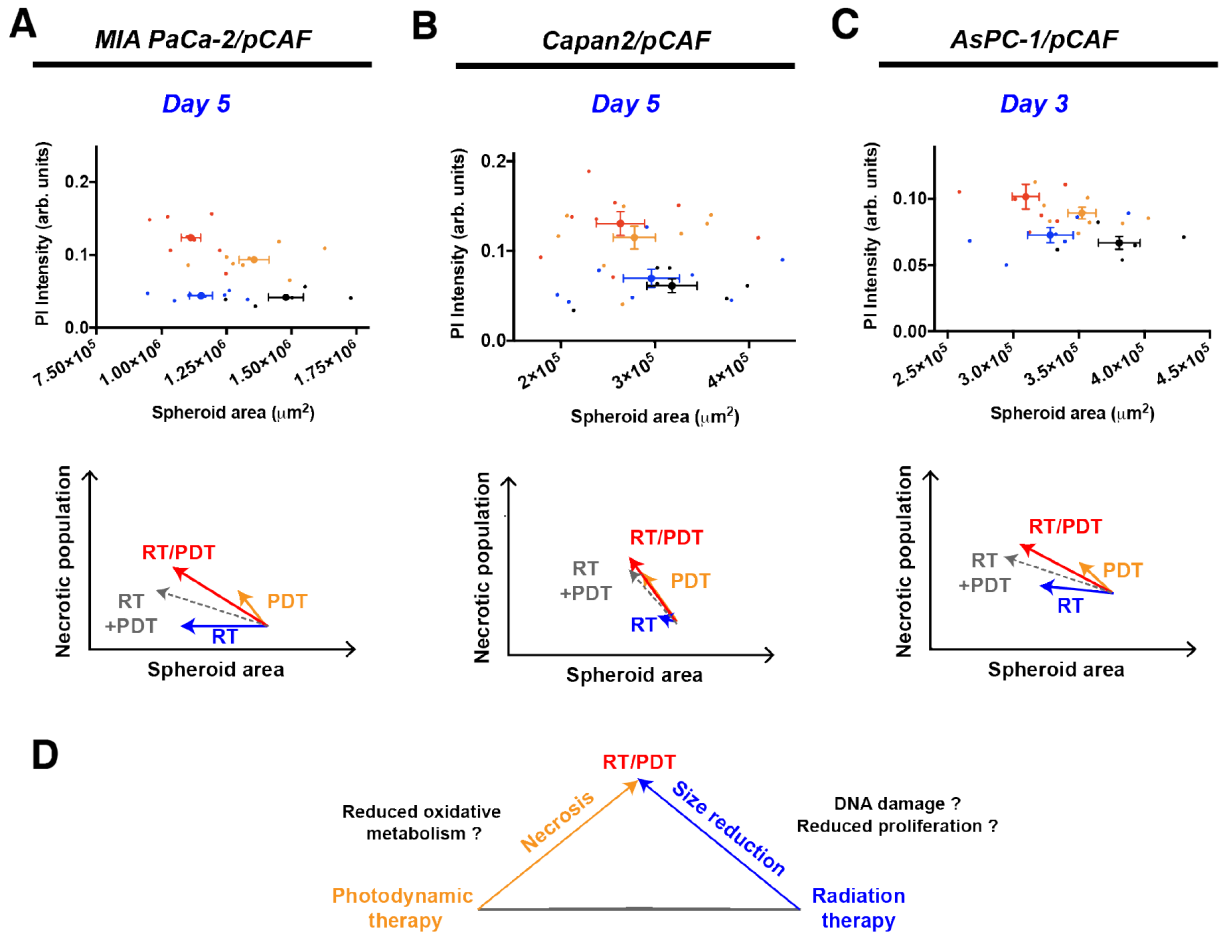
Correlation between the PI intensity, i.e. the necrotic content, and the area of the spheroids for MIA PaCa-2/pCAF **(A)**, Capan2/pCAF **(B)** and AsPC-1/pCAF **(C)** cultures. For each culture type, four conditions are represented: the untreated control group (black), 2.5J/cm^2^ PDT (orange), 20Gy RT (blue) and the combination of 2.5J/cm^2^ PDT with 20 Gy RT (red). For each condition, individual spheroids are represented by a thin scatter whereas a thicker scatter is used to represent the mean (+/- SEM) for each treatment group. The geometric sum of each effect is represented by a dashed grey arrow that represents the direct sum of the RT and PDT effects. In **(D)** is represented a schematic drawing of the effect of each individual treatment, emphasizing that a RT/PDT combination leads to smaller and more necrotic spheroids.

Figure 4 depicts the PI intensity of each spheroid plotted as a function of its area for a selected day (day 5 for MIA PaCa-2/pCAF and Capan2/pCAF co-cultures and day 3 for AsPC-1/pCAF co-cultures). As already illustrated on Figures 2 and 3, RT is mainly responsible for the size decrease whereas PDT induces necrosis. Thus, when combining RT with PDT, we obtain smaller and less viable spheroids (Figure 4D). In addition, when summing the effects of RT and PDT using a geometrical representation, we obtain the grey arrows depicted on the Figure 4A, 4B and 4C. When comparing those grey arrows with the red ones that correspond to spheroids that received both RT and PDT, we notice that for each culture type, the necrotic population measured is higher than expected by the simple addition of RT and PDT. Regarding the size reduction, the combination RT/PDT induces a stronger size reduction than any individual treatment. However, the size reduction is slightly smaller than the one expected by summing the effect of RT and PDT. Finally, it has to be noted that the intensity of this effect varies from a model to another although the trend remains the same: the spheroid size reduces more when PDT and RT are applied than for each individual treatment, yet slightly less than the addition of the individual effect of each therapy, whereas PDT and RT synergistically enhance the necrosis in the treated co-cultures.

### The beneficial effects of PDT on the RT efficacy stem from reduced proliferation as well as increased apoptosis and DNA damage

Although tumor size and extent of necrosis are valid markers of treatment response, they do not take into account potential treatment effects on viable cells. BPD-PDT demonstrated the capacity of inducing direct cell death through necrosis, yet it is also a potent inducer of apoptosis through various mechanisms [12, 66]. Similarly, RT is a potent inducer of apoptosis through the activation of DNA damage that could either lead to cell death or to a cell cycle arrest. This cell cycle arrest could be either temporary to allow the cells to repair their DNA or permanent in case of an unsuccessful repair [58]. Thus, to explain the effects of PDT, RT, and the PDT-RT combination on the spheroid size and extent of necrosis, we additionally investigated the expression of the proliferating cell nuclear antigen (**PCNA**) and of the DNA damage marker phospho-histone 2A-X (**γ-H2AX**) in the different PanCa spheroid models.

**Figure 5:**
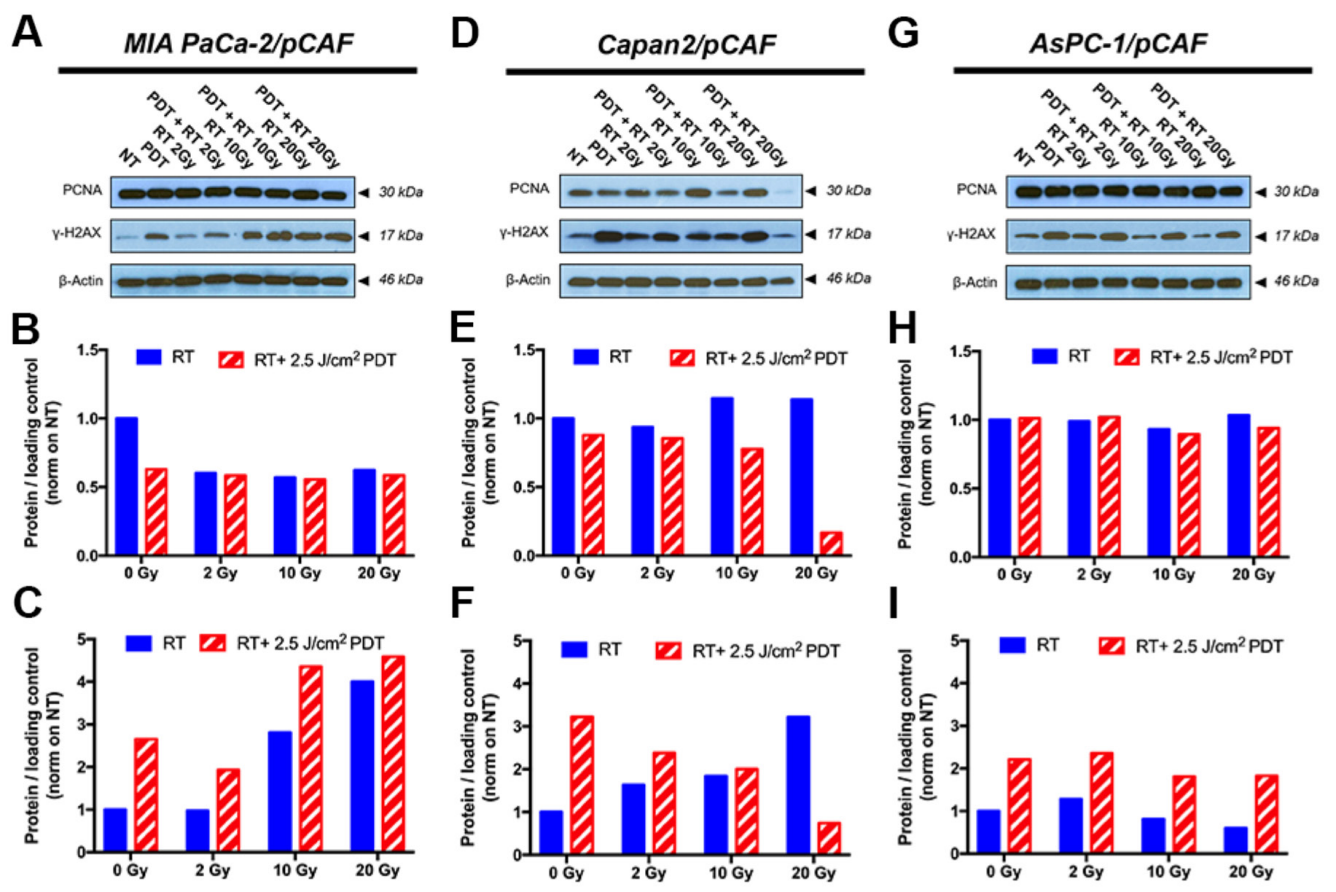
Representative immunoblotting performed on samples collected on day 5, i.e. 2 days post-treatment, are displayed on figures **(A**, **D** and **G)** and allow to assess the expression level of PCNA and *γ*-H2AX; *β*-actin is used as a loading control. Quantification of the PCNA band intensity is reported on graphs (**B**, **E** and **H**) whereas the quantification of the *γ*-H2AX band intensity is depicted on figures **(C**, **F** and **I)** for the selected blots. The data pooled from the repeats are presented in Supplementary Figure 4 and corroborate the results presented here. The results are presented for each culture type: MIA PaCa-2/pCAF **(A**-**C)**, Capan2/pCAF **(D**-**F)** and AsPC-1/pCAF **(G**-**I)**.

**Figure 6:**
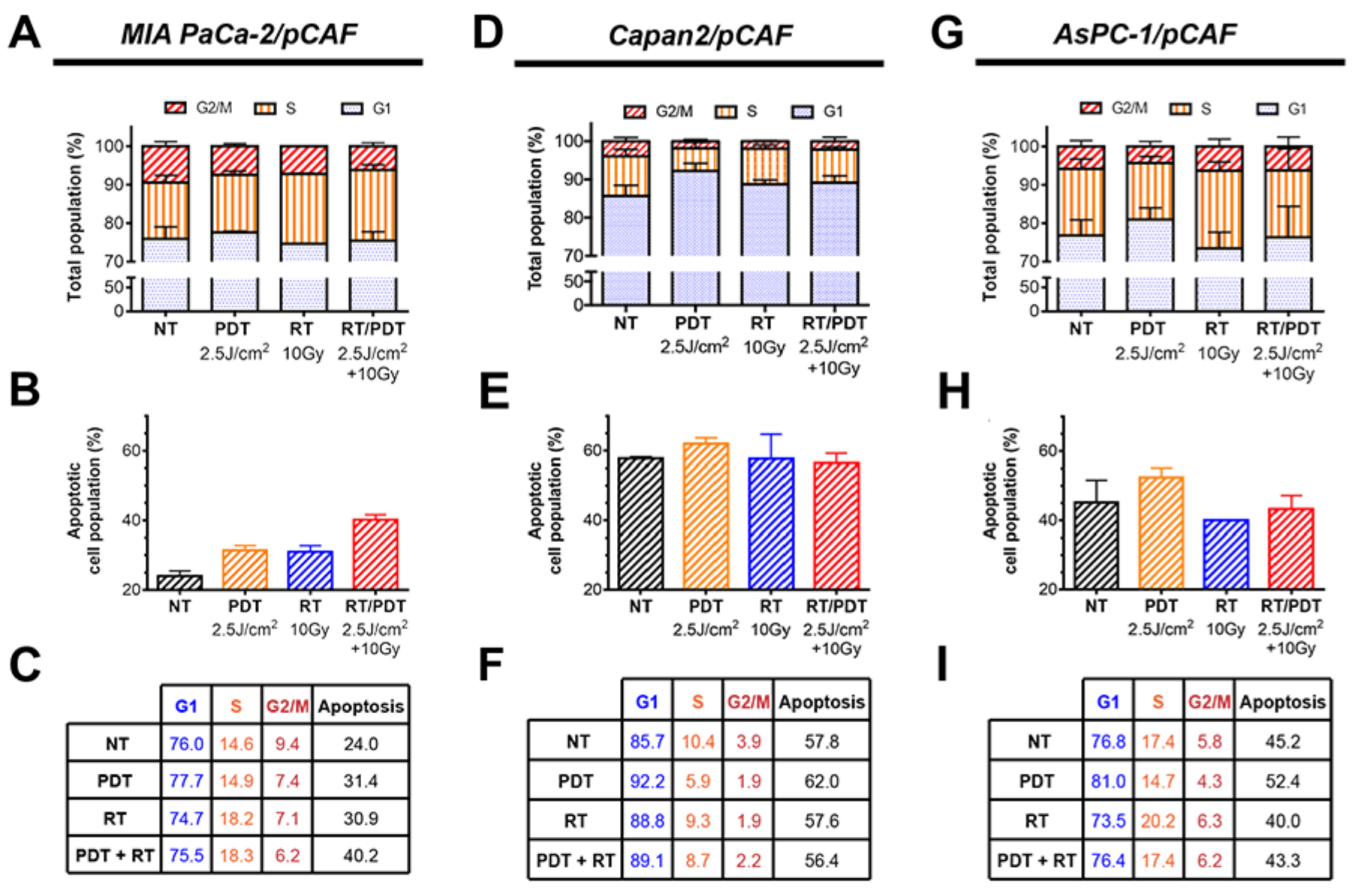
**(A**, **D** and **G)**. Cell cycle distribution assessed by flow cytometry using PI-stained cells obtained after spheroids were collected, disrupted and cells were fixed on day 8 (i.e. 5 days post-treatment). The percentage of apoptotic population present amongst the spheroids is also represented on the graphs **(B**, **E** and **H)**. All the values are reported (in percentage) in the tables **(C**, **F** and **I)**. All the results are presented for each culture type: MIA PaCa-2/pCAF (**A**-**C**), Capan2/pCAF **(D**-**F)** and AsPC-1/pCAF **(G**-**I)**.

Regarding the expression of γ-H2AX, all three spheroid models constitutively express low levels of this DNA damage marker. Similarly, PDT alone induces a slight over-expression of this marker in all three models. As for the effects of RT and RT/PDT combinations, the results vary for each cell types as will be discussed below. When investigating the PCNA expression, we observed that none of the treatments decreases the PCNA expression levels in MIA PaCa2/pCAF cultures. Knowing that the PCNA protein expression is directly related to the proliferation speed of the cells [67], the results suggest that the viable cells proliferate at a similar rate, regardless the treatment (Figure 5A, 5B and Supplementary Figure 4B). With respect to γ-H2AX, in this model the amount of γ-H2AX expression is increased by RT in a dose-dependent manner. When RT is given in combination with PDT, the expression of γ-H2AX is slightly enhanced compared to the expression of γ-H2AX for RT-treated samples (Figure 5A, 5C and Supplementary Figure 4A). Investigation of the cell cycle reveals that both PDT and RT increase the apoptotic population: while 24% of the cells are undergoing apoptosis in the untreated control group, this percentage goes up to 31.4% for PDT only, to 30.9% for 10Gy RT, and to 40.2% for the RT/PDT combination (Figure 6A–6C).

In the case of the Capan2/pCAF cells, much more noticeable effects appear: while PDT alone appears to cause a mild reduction in the PCNA expression, this latter increases with RT in a dose-dependent fashion. However, when combining PDT with an increasing dose of RT, PCNA expression is strongly diminished (Figure 5D, 5E and Supplementary Figure 4D), emphasizing that the remaining cells proliferate much slower than when they remain untreated. For γ-H2AX, the expression of this DNA-damage marker increases with the RT dose when RT is given alone whereas it decreases with the RT/PDT combination (Figure 5D, 5F and Supplementary Figure 4C).

Finally, for the AsPC-1/pCAF cultures, while neither PDT nor RT alone reduce PCNA expression, a slight decrease in PCNA levels are observed when PDT is combined with 10 Gy or 20 Gy RT (Figure 5G, 5H and Supplementary Figure 4F), demonstrating a mild proliferation reduction. As for the AsPC-1/pCAF cells, the expression level of γ-H2AX decreases when the RT dose increases, whereas this reduction is not as distinct when RT is combined with PDT (Figure 5G, 5I and Supplementary Figure 4E).

To strengthen the results obtained about the cell proliferation, we performed cell cycle profiling of the remaining populations. Therefore, we investigated the extent of PDT, RT, and PDT-RT to induce apoptosis, and/or cell cycle abnormalities. Regarding the cell cycles, we observe that low dose PDT increases the G1 phase in all culture types (Figure 6), although the increase is very mild for the MIA PaCa-2/pCAF spheroids (Figure 6A, 6C). The S population drops between the untreated (*NT*) and PDT groups for Capan2/pCAF and AsPC-1/pCAF spheroids showing that the cells slow their DNA replication mechanisms. However, this population remains constant for the MIA PaCa-2/pCAF cultures, which demonstrates that the remaining cells keep replicating with an identical rate as if they were untreated. Lastly, low-dose PDT causes a decrease in the G2/M populations regardless the culture type.

When applying RT at 10Gy, we observe an increase in the G1 phase for the Capan2/pCAF spheroids, as well as a decrease in the S phase population, whereas an opposite situation is reported for the MIA PaCa-2/pCAF and AsPC-1/pCAF cultures where the G1 population decreases and the S population increases. As for the G2/M population, it drops for both MIA PaCa-2/pCAF and Capan2/pCAF spheroids but slightly increases for the AsPC-1/pCAF spheroids.

When combining the two therapies, various results are observed depending on the culture type. While the AsPC-1/pCAF cells present a similar distribution compared to the untreated group (Figure 6G, 6I), the MIA PaCa-2/pCAF cultures present a slight decrease in the G1 population, a 3.7% increase in the S population and a 3.2% decrease in the G2/M population regarding the untreated control group (Figure 6C). As for the Capan2/pCAF populations, we report a 3.4% increase in the G1 population and a 1.7% decrease in the S and G2/M phases (Figure 6F).

Taken together, analysis of the cell cycle profiles suggests that while Capan2/pCAF cultures are arrested in the G1 phase where they can repair their DNA impaired by treatment assault, MIA PaCa-2/pCAF and AsPC-1/pCAF cultures keep synthesizing their DNA and proliferating. This distinction is in agreement with the fact that a defect in p53 prevents the G1 arrest after RT for DNA repair [68].

In conclusion, the cell cycle profile data support the expression levels of PCNA, suggesting that RT induces no significant reduction in proliferation rates for the remaining cell population. In contrast, PDT induces reduced proliferation by restraining cells in the G1/G0 phase, an effect best observed in the Capan2/pCAF model. The expression levels of γ-H2AX suggest that BPD-PDT induces DNA damage, as this effect was uniformly observed in all three models. Although BPD-PDT is known to be incapable of inducing DNA damage directly [9, 12, 69–71], the increased levels of γ-H2AX likely stem from the degradation of DNA during apoptosis [72]. For MIA PaCa-2/pCAF spheroids, the adjuvant beneficial effect of low-dose PDT on RT is reflected in its capacity to enhance levels of apoptosis, necrosis, resulting in reduced tumor sizes. For Capan2/pCAF and AsPC-1/pCAF spheroids, the adjuvant effects are similarly established, but also include a PDT-induced cell cycle arrest in the G1 phase.

### Optical redox ratio correlates with treatment induced necrotic population

It has been demonstrated that the efficiency of RT can be enhanced by a prior BPD-PDT treatment in fibrosarcoma cells [73]. In this study, it was shown that non-vascular BPD-PDT could decrease the oxygen consumption of the cancer cells *in vivo* by slowing down mitochondrial respiration, while preserving the vasculature. Through those two mechanisms, the oxygen availability increases within the tumor, thus enhancing the efficacy of a subsequent RT by making the DNA damage more permanent. To investigate this effect, one can measure the reduced nicotinamide adenine dinucleotide (**NADH**) and oxidized flavoprotein adenine dinucleotide (**FAD**) fluorescence signals, as these endogenous fluorophores that are mainly present within the mitochondria are related to the respiratory status of a culture [74]. In order to quantify the amount of NADH and FAD within a culture, the optical properties of these endogenous species can be leveraged [75–77]. In this study, we utilize a methodology that we adapted from those techniques to non-disruptively assess the redox state of PanCa spheroids to be published.

The three PanCa cell lines that have been used in this study are known to express various metabolic phenotypes: while MIA PaCa-2 cells are mainly glycolytic, AsPC-1 and Capan2 have been reported to have a “senescent phenotype” that involves higher activity of mitochondrial respiration [78].

**Figure 7:**
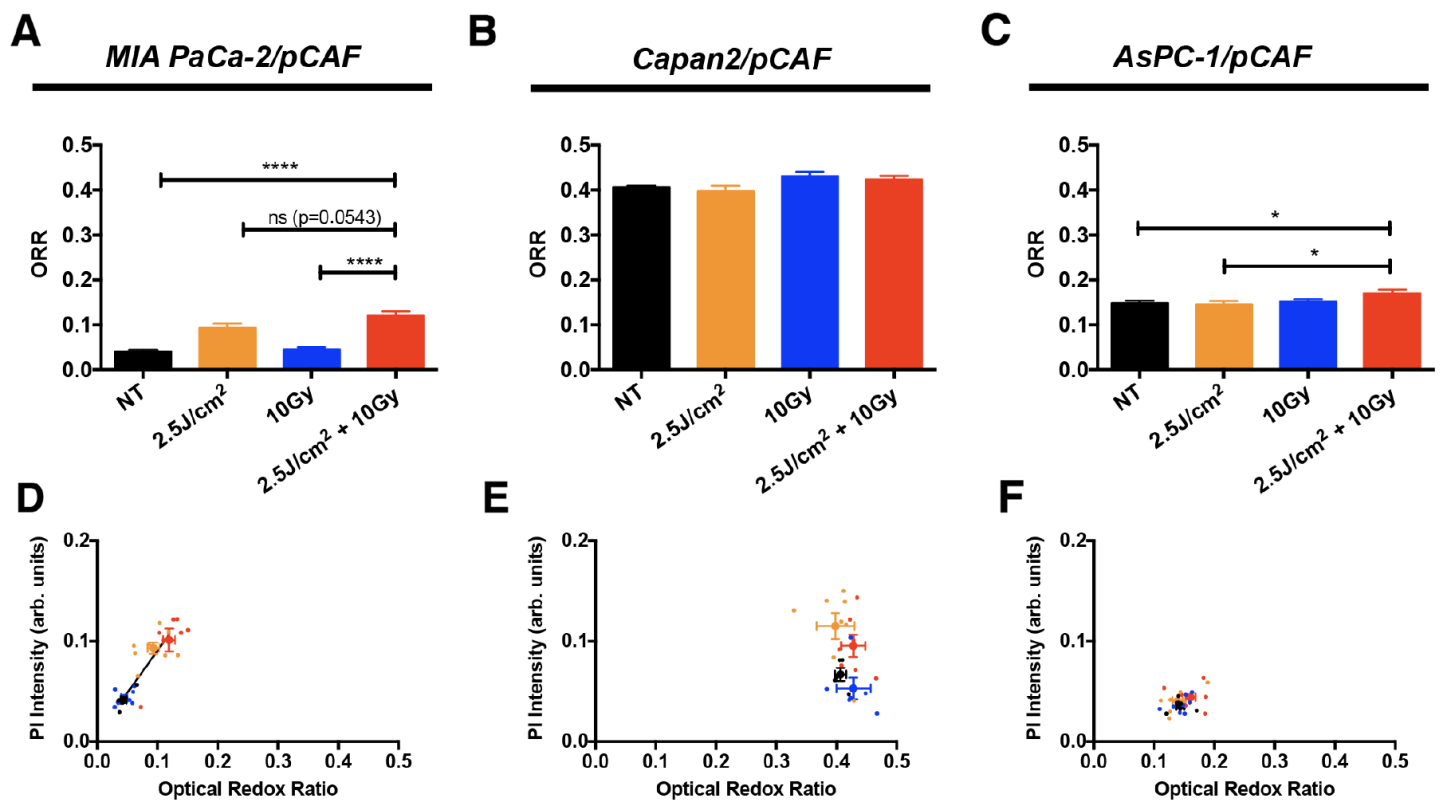
The ORR (mean +/- SEM) has been assessed on day 5, i.e. 2 days post-treatment, on untreated spheroids and spheroids that received 2.5 J/cm^2^ of PDT, 10 Gy of RT or a combination of 2.5 J/cm^2^ of PDT and 10 Gy RT, for **(A)** MIA PaCa-2/pCAF cultures, **(B)** Capan2/pCAF and **(C)**. AsPC-1/pCAF. A correlation between the size of the spheroids and their ORR was represented for the same cultures: **(D)** MIA PaCa-2/pCAF cultures, **(E)** Capan2/pCAF and **(F)** AsPC-1/pCAF; where the untreated controls are represented in black, the PDT treated samples in orange, the spheroids that received 10Gy RT are in blue and the cells that received a combination of RT/PDT are depicted in red. For each treatment group, each spheroid is represented by a small scatter and the average value (mean +/- SEM) calculated for each treatment group is represented by a thicker point.

According to the theory of Pogue et al. [73] and the metabolic phenotyping by Daemen et al. [79], we should expect to see the biggest differences in the AsPC-1/pCAF spheroids, as these rely the most on oxidative phosphorylation (OxPhos). Alternatively, given that basal redox states are substantially higher in the Capan2/pCAF spheroids, it may be expected that oxygen consumption is highest in this model (Figure 1D, 1E). However, the most notable difference in redox state were observed in the MIA PaCa-2/pCAF model (Figure 7A, 7D), in which the PanCa cells supposedly relied mostly on reductive glycolysis. These results therefore did not support the hypothesis that the enhanced RT efficacy by PDT stems from reduced oxygen consumption by PDT. However, perturbations in redox state can stem from various origins, including metabolic shifts, oxidative stress, and mitochondrial uncoupling prior to apoptosis. We therefore hypothesize that the observed elevated redox states in the MIA PaCa-2/pCAF spheroids, and to a lesser extent in the AsPC-1/pCAF model, is likely to originate from one or more of these factors. Indeed, there appeared to be a positive correlation between the redox state and the extent of necrosis for the MIA PaCa-2/pCAF spheroids (Figure 7D). However, similar correlations were not found for the other PanCa models (Figure 7E–7F).

## DISCUSSION

With the aim of evaluating the efficacy of low dose PDT with RT in relevant models, we first developed spheroids models of PanCa. Given that these cancers are hallmarked by a dense stroma containing fibroblasts that can affect treatment responses through protective encapsulation or providing metabolic support [49, 79, 80], we considered that the presence of pCAFs was necessary for the accurate evaluation of treatment effects. To account for PanCa heterogeneity, we used genotypically and phenotypically distinct pancreatic cancer cells as basis for these models. Using these heterotypic models, we demonstrated that low-dose PDT exerts a beneficial effect on the overall response to RT. In a general way, we observe that RT uniformly reduced tumor sizes while low-dose PDT enhanced the extent of necrosis, resulting in smaller and less viable spheroids, as summarized in Figure 4. However, the treatment effects and their timing differed substantially between the different tumor models. Although the PDT-induced necrotic response was universal across the different pancreatic tumor models, the molecular and cellular effects of the RT/PDT treatment were more divergent across the tumor models, as further discussed below and the model originated from metastatic cancer cells appears to be less sensitive to treatment.

First, the interpretation of spheroid areas was challenged by variations in spheroids structures and required a careful analytical approach. Indeed, in the case of MIA PaCa-2/pCAF cultures that form dense and homogeneous spheroids, the area directly resembles the treatment effect on the culture. Therefore, we demonstrated that RT controls the tumor size and that PDT, when combined to RT, induces an additional size decrease. On the contrary, spheroid cultures composed of Capan2/pCAF and AsPC-1/pCAF consisted of a core and a halo of loose cells. When reporting the total area (core+halo), a growth can mean either a growth of the core and/or a spread of the surrounding cells. In these two culture models, a careful analysis of the images along with a quantification of the area confirmed that the size of the spheroid core decreases while the overall area increases, highlighting an amplified spread of the surrounding cells (Supplementary Figure 3). Although beyond the scope of this research, these observations may indicate loss of cell-cell adhesions and loss of overall spheroid integrity by the treatments, but may also indicate increased cell migration.

Secondly, investigations of the PCNA expression level by immunoblotting, cell cycle profile and apoptotic population of the remaining cells revealed fundamental differences between cells that express a wild type p53 status (Capan2) and cells that are p53 mutated (MIA PaCa-2 and AsPC-1): when p53 is functional, the cells tend to slow their proliferation down and accumulate in the G1 phase after RT to repair their DNA, whereas the cells that have a p53 mutated phenotype keep proliferating at a normal rate. γ-H2AX levels revealed remaining unrepaired DNA double-strand breaks 2 days post-treatment (Figure 5). This marker is usually measured within a few hours following the treatment to reveal DNA-damage [81]. However, phosphorylated H2AX remaining more than 24 hours after RT can be a sign of radioresistance as it indicates unrepaired or incorrectly repaired DNA double-strand breaks [60, 82]. Taken together, the apparent differences in treatment effects on spheroids derived of the different, yet equally clinically relevant pancreatic cancer cell types underscore the importance of assessing complex combination therapies on multiple models. In the context of this study, comparing these models highlights the fact that while the low dose PDT/RT combination leads to smaller and less viable spheroids, the effects of individual or combination therapies on the remaining populations may vary and induce for instance, various phenotypes, unrepaired DNA-damage or modifications in the spheroid architectures.

Previously, the combination of RT with conventional PDT has been investigated in a plethora of models, and with multiple different conditions (e.g. sequence order, delay between the two treatments, tumor models, *in vitro* versus *in vivo*). Back in 1955, Schwartz et al. already investigated the combination of hematoporphyrin with X-rays on patients diagnosed with various types of cancer to validate the effect of PDT in sensitizing a tumor to a subsequent RT [83]. However, the results were not as categorical as expected and more than sixty years later, there is still no unequivocal answer about the effect of combining RT with PDT. Additive effects have been reported *in vitro* [84–91]. Synergistic effects have also been reported, mainly *in vivo*, and although mechanisms involving tumor reoxygenation were proposed to explain those observations, no consensus has yet been reached [92–94]. In addition, it was demonstrated that the delay between the PDT and the subsequent RT is critical to turn an additive effect into a synergistic one [89, 90, 94]. It has been shown that when the PS accumulates in the tumor, an initial PDT can briefly improve the tumor oxygenation and improve the effect of a subsequent RT [94]. However, to benefit from the brief reoxygenation induced by the PDT, it was shown that the RT should be given simultaneously or a very short period of time after the PDT [73]. However, our findings could not corroborate this phenomenon, which is explained by a prolonged time-delay between PDT and RT in our study. The two therapies were given with a 10 minutes interval during which the tumor cultures were transported from the PDT irradiation platform to the X-ray generator. As such, the effects of PDT on the oxygenation status of the tumor spheroids may have normalized during this time interval. Despite this, we still observed a beneficial effect of PDT on the RT efficacy that likely emerged from a different mechanism. Together, these findings strongly support further development of nanoscintillator-induced deep-tissue PDT application, where both therapies are simultaneously activated by the ionizing radiations.

In the context of RT-induced deep-tissue PDT using nanoscintillators, both Monte Carlo simulations [35, 36] and experimental studies [37] conclude that the PDT dose that will be induced will be very low. In order to estimate whether these doses would be strong enough to induce cell death, an estimation of the number of singlet oxygen molecules that would be generated was provided and compared to the minimal amount of singlet oxygen molecules that have to be produced to induce cell death. Most of those studies use as a comparison one of the less restrictive value found in the literature that was proposed by Niedre *et al.* [95], although other values are reported [96]. Most of those studies conclude that the amount of singlet oxygen molecules that will be generated is too low to induce cell killing, yet we demonstrated that low dose PDT harmonized with RT and achieved encouraging results to pursue nanoscintillators-induced deep tissue PDT.

The therapeutic effects of deep-tissue PDT using novel nanoscintillator-PS conjugates are anticipated to encompass a unique combination of mechanisms. As nanoscintillators are typically composed of high-Z elements a combined treatment modality may achieve improved treatment outcomes by mediating four main contributions: the RT, the PDT, the slight beneficial effect obtained by combining RT with low dose PDT and finally a radiation dose enhancement (RDE) effect. This effect is caused by the presence of high-Z elements in the tumor during RT that will increase the tumor specific radiation dose deposition. In preclinical settings, this RDE effect remarkably improved one-year survival rates of mice bearing subcutaneous tumors (20% to 86%) [33]. We are currently working on investigating the impact of this latter effect on the overall treatment efficiency.

In conclusion, the biomedical exploitation of scintillating nanoparticles provides a groundbreaking approach to overcome the main limitation of PDT. The nanoscintillator-mediated excitation of photosensitizers utilizes deep-penetrating X-rays instead of low-penetrating near-infrared light, thus overcoming both the size and localization-dependent restrictions that currently limit the application of conventional PDT. As X-rays are inherently more toxic than visible light, the therapeutic exploitation of nanoscintillator-mediated PDT should be primarily focused on cancer types in which RT is involved in the standard of care. The findings of this study provide excellent preliminary evidence that low-dose PDT combined with RT effectively restrained pancreatic tumor growth while concomitantly increasing tumor necrosis. These findings encourage the development of therapeutic approaches that include the use of nanoscintillators to simultaneously induce RT and deep-tissue PDT. The envisioned therapeutic modality represents a mechanistically unique combination that may hold significant promise in the treatment of cancer types for which RT is involved in the current standard of palliative care, such as pancreatic cancer.

## MATERIALS AND METHODS

### Cell culture and reagents

Pancreatic cancer cell lines MIA PaCa-2, AsPC-1, and Capan2 were obtained from the American Type Culture Collection (ATCC, Manassas, VA). Pancreatic cancer-associated fibroblasts (pCAF) were isolated from primary pancreatic tumors as described previously [56]. All four-cell lines were maintained in DMEM supplemented with 10% (v/v) FBS, 5mM glutamine, and 1% (v/v) penicillin/streptomycin. All cell lines were typically passaged weekly at a 1:8 ratio and maintained at standard culture conditions (37°C, 5% CO2). Throughout the experiments, all lines were confirmed mycoplasma free as assessed using the MycoAlert Plus mycoplasma detection kit (Lonza, Portsmouth NH).

### 3D cultures

The tumor spheroids were grown in ultra-low adhesion 96-well plates (Corning). Cells were seeded at a density of 5000 cells/well: 2500 cells/well of PanCa cells (50μL of a 50000 cells/mL suspension) and 2500 cells/well of pCAF (50μL of a 50000 cells/mL suspension). To produce stroma-rich tumor models that resemble PanCa, we arbitrarily chose a 1:1 ratio (cancer cells:fibroblasts) as previously reported [97]. Indeed, as CAF can quickly outcompete cancer cells in a culture [98], we chose a 1:1 ratio rather than a higher ratio of fibroblasts.

### Tracking spheroid growth

Spheroids were imaged daily using a Zeiss Axiovert microscope (Thornwood, NY). Spheroid areas were extracted from the bright-field images using a custom-developed Matlab code, which uses adaptive thresholding to binarize each image and outline the spheroids. For both AsPC-1/pCAF and Capan2/pCAF co-cultures, spheroids displayed a typical morphology associated with cells migrating from the core. For these cultures the code was run twice with different thresholding sensitivity to isolate both the spheroid cores and the halo of migrating cells.

### Tracking spheroid composition

To distinguish and track the cancer cells and fibroblasts in the spheroid cocultures, each cell population was fluorescently labeled with quantum dots (**QD**) for longitudinal tracking of spheroid compositions. Two types of QD were used: one with a maximum emission at 525 nm, the other one at 655 nm (QTracker kits, ThermoFisher Scientific, Cambridge, MA), both excited using a.

### Cell staining protocol

Before initiation of the co-cultures, each cell line was individually labeled with 20nM QD. Briefly, cells were harvested from the culture flasks and 1×10^6^ cells/mL suspensions were prepared. The staining protocol was a two-step process: first the required volume of QD (component A of the kit) was mixed with the same volume of buffer solution (component B of the kit) in a 2mL tube and incubated at room temperature for 5 minutes. Then, the chosen volume of 1×10^6^ cells/mL was added to the QD staining solution; the solution was homogenized and placed in the incubator for (37°C, 5% CO_2_) for 30 minutes. Finally, the cells were washed thrice to remove the non-internalized QD. Subsequently, the cells were counted with a hemocytometer using the trypan blue exclusion assay (ThermoFischer Scientific). The individually labeled cell solutions were diluted to prepare a 5×10^4^ cells/mL and were seeded as previously described.

### Imaging protocol

A two-photon 790nm pulsed excitation was generated using a MaiTai Deep See red/IR tunable laser (Spectra-Physics, Santa Clara, CA) and images were recorded using an Olympus FV1000 multi-photon confocal microscope mounted with a 10X air-objective (0.4 NA). 512×512 px images were acquired with an integration time of 12.5μs/px. Z-stacks were recorded with a 5μm z-axial resolution. The signal emitted by the 525nm- and the 655nm-QD were collected through a 520±20nm and 650±20nm filter respectively.

### Treatment delivery

All treatments were initiated on the third day of culture. PDT was performed by incubating spheroids for 1 hour with 0.25μmol/L benzoporphyrin-derivative (**BPD**) in culture medium. After incubation, each well received the indicated light dose (ranging from 0.5-40J/cm^2^ as indicated) delivered by a 690nm laser (Intense Ltd. North Brunswick, NJ) at a controlled irradiance of 150mW/cm^2^. RT was performed using an X-rad 320 irradiator (320kV, 12.5mA). The X-ray radiation was given to the spheroid cultures through a 2 mm aluminum filter at a fixed dose rate of 2.75Gy/min.

### Viability assessment

Quantitative determination of treatment outcomes on the 3D cultures was performed using *in situ* staining of the spheroids with 2μM calcein AM and 3μM of propidium iodide, followed by imaging using confocal laser scanning microscopy (Olympus FV1000), after which image analysis was performed according to the CALYPSO methodology for multiparametric assessment of treatment effects as described previously [4]. More technical details could be found in the Supplementary Materials (SI1).

### Redox imaging

The optical redox ratio [75–77] was determined by imaging the autofluorescence of nicotinamide adenine dinucleotide (NADH) and flavoprotein adenine dinucleotide (FAD) using 2-photon excited fluorescence microscopy. The spheroids were imaged using a multiphoton confocal microscope (Olympus FV1000) through a 10X objective (0.4NA, air). The 750nm pulsed excitation was delivered by a tunable MaiTai DeepSee red/IR laser unit and the autofluorescence signals were acquired using two band-pass filters: 440±20nm and 520±20nm for NADH and FAD respectively. The optical redox ratio (**ORR**), defined as the ratio of the FAD autofluorescence signal divided by the sum of the FAD and NADH fluorescence signals, quantified using a custom-written script in Matlab 2016B (Mathworks, Natick, MA).

### Immunoblotting

Spheroids were pooled (8 wells), washed in PBS, and lysed in RIPA buffer supplemented with protease inhibitor cocktail (Calbiochem) and phosphatase inhibitor cocktails I, II, and III (Sigma Aldrich). Protein concentrations were determined using the bicinchoninic acid assay (ThermoFisher). Protein samples (10μg) were electrophoretically separated on 4-20% Mini-Protean TGX gels (BioRad) and blotted on PVDF membranes (200 mA, 2h). Membranes were blocked for 1h in 0.1% Tween in Tris-buffered saline (TBST) supplemented with 5% BSA. Primary antibody incubation was performed overnight at 4°C in TBST with 5% BSA. Membranes were washed four times in TBST and incubated with secondary antibody in TBST with 5% BSA for 1h at room temperature. Subsequently, membranes were washed four times in TBST, and protein levels were detected using clarity enhanced chemoluminescence kit (BioRad), and CL-Xposure radiographic films (ThermoFisher). All immunoblots are representatives for at least 2 independent experiments. Antibodies details: anti-PCNA (Cell Signaling, 2586, dilution 1:1000), anti-γ-H2AX (Cell Signaling, clone 20E3, dilution 1:1000), anti-β-Actin (Sigma Aldrich, clone AC-74, dilution 1:5000). The secondary antibodies: goat-anti-mouse (Cell Signaling) or goat-anti-rabbit (Cell signaling, 70475) were conjugated to horseradish peroxidase and used with the dilution ratio 1:1000.

Quantification of the Western blots was performed using the ImageJ software (version 2.0.0). For each protein, the scanned image of the blot was converted into a grayscale image. A region of interest (ROI) was defined and the mean intensity of each band was extracted. The background was similarly obtained by measuring the mean intensity in the same ROI surface positioned right above or below the considered band. After inverting the intensity (255-measured intensity), the difference (Inverted Signal – Inverted background) was calculated. This calculation was performed for each protein including the loading control and data was normalized on the no treatment condition.

### Flow cytometry

Cell cycle profiling was performed by dissociation of the tumor spheroids. For each sample, 8 spheroids were pooled, washed in 2.5mL of PBS and centrifuged for 5min at 500×g. The supernatant was aspirated and the spheroid pellet was resuspended in 1mL Cellstripper (Corning). After 10 minutes incubation, the solution was centrifuged for 5min at 500×g, the supernatant was aspirated and the cells were resuspended in 300μL PBS and stored on ice. Cell fixation and PI staining was performed as previously described [99]. Cells were fixed by adding 700μL ethanol (-20°C) in a drop-wise fashion under constant swirling. The fixed cells were centrifuged and the supernatant was aspirated. The permeabilized cells were incubated for 30 minutes at 37°C in 100μL PBS containing 50μg/mL PI and 20μg/mL RNAse A. Cell cycle profiling was performed using a FACS ARIA III (Becton Dickinson, Waltham, MA), using λ_exc_= 561 nm and λ_em_= 610±20 nm, recording 5000 events/sample.

### Statistical analysis

All statistical analysis was performed using GraphPad Prism 7.0 (La Jolla, CA). Data sets were checked for normality using the d’Agostino Pearson omnibus normality test. Student *t*-test or one-way ANOVA followed by a Bonferroni post hoc test were then used to assess statistical significance.

## SUPPLEMENTARY MATERIALS FIGURES



## Abbreviations

BPD: benzoporphyrin derivative
FAD: flavoprotein adenine dinucleotide
NADH: nicotinamide adenine dinucleotide
ORR: optical redox ratio
PanCa: pancreatic cancer
PBS: phosphate buffer saline
pCAF: pancreatic cancer associated fibroblasts
PCNA: proliferating cell nuclear antigen
PDT: photodynamic therapy
PS: photosensitizer
QD: quantum dots
RDE: radiation dose enhancement
RMS: reactive molecular species
RT: radiation therapy
TBST: tween in tris-buffered saline
