# Supplementary material for: Low dose photodynamic therapy harmonizes with radiation therapy to induce beneficial effects on pancreatic heterocellular spheroids
Source: Oncotarget. 2019 Apr 5;10(27):2625–43. doi: 10.18632/oncotarget.26780 (PMC6499000; doi:10.18632/oncotarget.26780)
Supplement: Supplementary file 1 [file oncotarget-10-2625-s001.pdf]

Low dose photodynamic therapy harmonizes with radiation therapy to induce beneficial effects on pancreatic heterocellular spheroids

SUPPLEMENTARY MATERIALS

SI1: Experimental details about the calcein AM/propidium iodine viability assay

The 3D culture viability was assessed using a live/dead fluorescent-based assay (Calcein AM and Propidium iodide (**PI**) used to label live and necrotic cells respectively) [1, 2]. Images were acquired using a Olympus FV1000 confocal laser scanning fluorescence microscope and were further processed using the CALYPSO image analysis procedure previously described [1]. Briefly, the volume of each well was brought down to 50μL and 50μL of the staining solution was added to each well. The staining solution was prepared with twice the desired concentration in calcein AM and PI to reach the desired concentration in the well: 2μmol/L calcein (staining solution made with 4μmol/L) and 3μmol/L PI (staining solution made with 6μmol/L) diluted in phosphate buffer saline. The spheroids were incubated with the staining solution for 60 minutes before being imaged on the confocal microscope. The fluorescence signals of the calcein AM (λ_{em}=500-525 nm) and PI (λ_{em}=600-660 nm) were recorded upon an excitation of 488 nm and 559 nm respectively through a 4X objective (0.16NA, air). The images were processed to extract the viability defined as the ratio of the live signal divided by the sum of the live and dead signals as well as the PI intensity, on a pixel-by-pixel manner using the CALYPSO image processing routine that was previously described [1]. The live and dead signals dynamic ranges were fixed using the no treatment control and a total killing control respectively. The total killing (**TK**) control was prepared right before incubating the culture with the calcein AM/PI mix [1, 2]. Briefly, spheroids designated to serve as TK control were fixed for 2 minutes using a 4% formaldehyde solution. A Triton X-100 solution was then added onto the fixed spheroids (0.5%) to permeabilize the membranes and simulate a fully necrotic spheroid. After 30 minutes incubation with the triton X, the spheroids were washed twice using a 0.1mol/L glycine solution. Those wells were then ready to receive the staining solution together with the treatment groups.

SI2: Selection of PDT and RT treatment parameters for establishing the RT/PDT combination

See Supplementary Figure 1.

SI3: Longitudinal radiation dose response

Although increasing doses of RT do not impair more the spheroid growth on day 5, if the area is measured on a later time point, we report a stronger size decrease for the MIA PaCa-2/pCAF model (Supplementary Figure 2). A non-linear regression was drawn when the fit could converge. As for Capan2/pCAF the relative size seems to be increasing. However, as previously described in the main document, the increase is more related to the spread of the cellular halo surrounding the core than an actual growth. Regarding the AsPC-1/pCAF spheroids, the changes are minor regardless of when the area is measured.

SI4: Investigation of the AsPC-1/pCAF growth

As previously discussed, the AsPC-1/pCAF spheroids exhibit a structure made of a dense core surrounded by a halo of cells. When analyzing the data, we can either select to measure the area of the entire cell population (core + halo), which is what is presented in the main manuscript, or we can isolate the area of the core only. We present on the Supplementary Figure 3 the results obtained when we consider the core and the halo. There, we notice that contrary to what was reported on day 12 in the main manuscript, the area of the core+halo of the spheroids increases after treatment. On the contrary, we report a mild increase induced by any type of treatment.

SI5: Semi-quantitative analysis of the western blots

See Supplementary Figure 4.

REFERENCES

1. Bardeesy N, DePinho RA. Pancreatic cancer biology and genetics. Nat Rev Cancer. 2002; 2:897–909. https://doi.org/10.1038/nrc949
2. Matsuoka L, Selby R, Genyk Y. The surgical management of pancreatic cancer. Gastroenterol Clin North Am. 2012; 41:211–21. https://doi.org/10.1016/j.gtc.2011.12.015

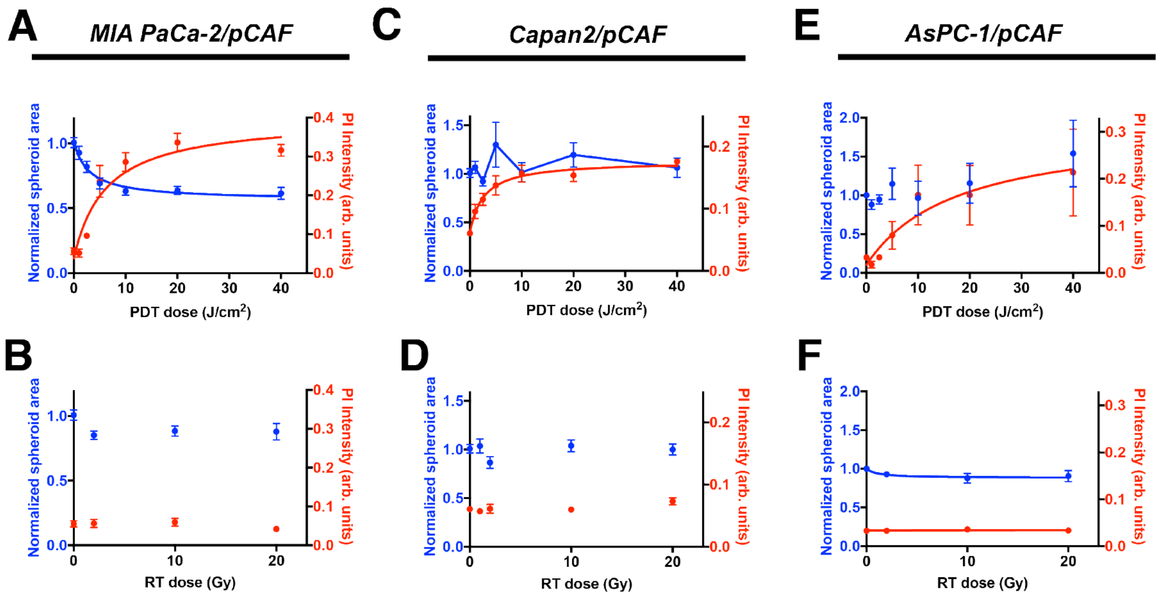

Supplementary Figure 1: Normalized spheroid area and average PI fluorescence intensity plotted as a function of the PDT light dose **(A, C** and **E)** or the radiation dose **(B, D** and **F)** for MIA PaCa-2/pCAF cultures **(A** and **B)**, Capan2/pCAF cultures **(C** and **D)** or AsPC-1/pCAF cultures **(E** and **F)**. A regression was also plotted when the fit was able to converge. In all three culture types, an increasing dose of PDT leads to a stronger PI intensity, emphasizing an increasing necrotic population. However, the treatment only impacts the size of the MIA PaCa-2/pCAF cultures and not of the AsPC-1/pCAF or Capan2/pCAF spheroids. The effects of raising doses of RT are mild both on the spheroid size and on the PI intensity.

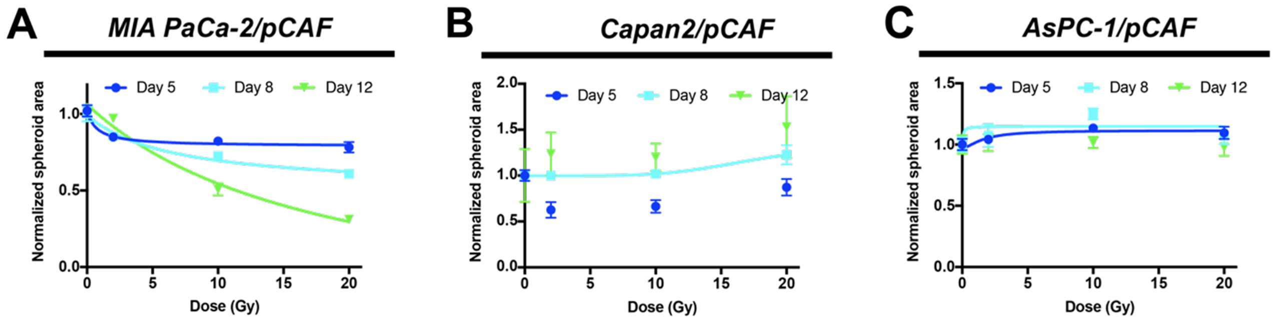

Supplementary Figure 2: Longitudinal RT dose response measured on each spheroid culture type: MIA PaCa-2/pCAF **(A)**, Capan2/pCAF **(B)** and AsPC-1/pCAF **(C)**. For each time point (day 5, day 8 por day 10), the averaged area are normalized on the no treatment condition. A regression was plotted along with the data point when a converging fit could be calculated. For the MIA PaCa-2/pCAF co-culture, the impact of the dose on the spheroid area increases with culture time.

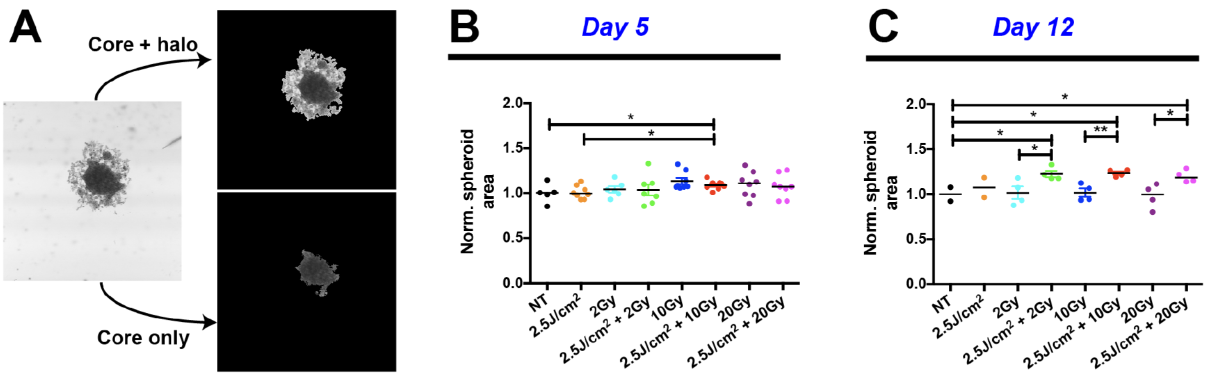

Supplementary Figure 3: **(A)** Illustration of the two possible ways of analyzing the spheroid area we compared: i) the core and the surrounding halo is considered (case we reported in the main manuscript) and ii) the core only. **(B** and **C)** Normalized area of the core of the AsPC-1/pCAF spheroids measured on day 8 (i.e. 5 days post treatment) and on day 12 (i.e. 9 days post treatment) respectively, after each treatment. The data demonstrate that compared to the results obtained and presented in the main manuscript, when the core and the halo of the spheroid is measured, the total area increases, emphasizing that the spread of the halo is enhanced.

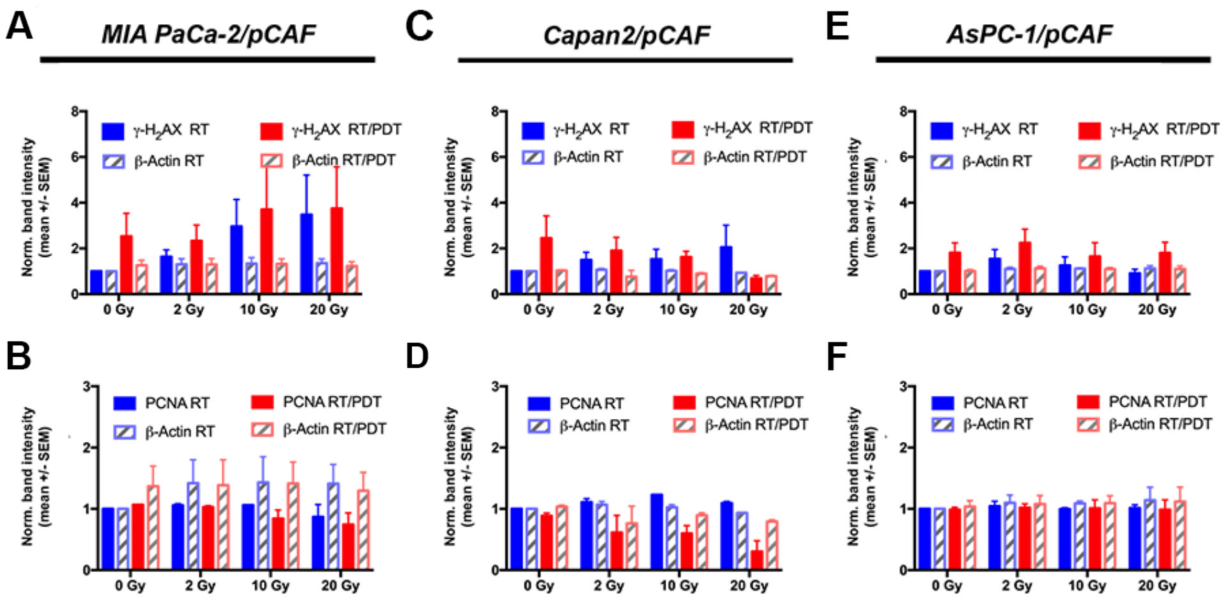

Supplementary Figure 4: Semi quantitative analysis of the Western blots for MIA PaCa-2/pCAF **(A, B)**, Capan2/pCAF **(C, D)** and AsPC-A/pCAF **(E, F)**. Panels **(A, C** and **E)** represent the normalized band intensity for the γ-H2AX and the loading control for samples exposed to an increasing dose of RT alone or to an increasing dose of RT combined with 2.5 J/cm^2 PDT, whereas panels **(B, D** and **F)** represent the normalized band intensity for PCNA together with the loading control. Data comes from at least two experimental repeats.
